# Enter the Matrix: Fibroblast-immune interactions shape ECM deposition in health and disease.

**DOI:** 10.12688/f1000research.143506.1

**Published:** 2024-02-19

**Authors:** Anthony Altieri, Grace V. Visser, Matthew B. Buechler

**Affiliations:** 1 Immunology, University of Toronto, Toronto, Ontario, Canada

**Keywords:** Fibroblasts, Fibroblast heterogeneity, Extracellular Matrix (ECM), Immunology, Macrophages, BigH3, Cthrc1

## Abstract

Fibroblasts, non-hematopoietic cells of mesenchymal origin, are tissue architects which regulate the topography of tissues, dictate tissue resident cell types, and drive fibrotic disease. Fibroblasts regulate the composition of the extracellular matrix (ECM), a 3-dimensional network of macromolecules that comprise the acellular milieu of tissues. Fibroblasts can directly and indirectly regulate immune responses by secreting ECM and ECM-bound molecules to shape tissue structure and influence organ function. In this review, we will highlight recent studies which elucidate the mechanisms by which fibroblast-derived ECM factors (e.g., collagens, fibrillar proteins) regulate ECM architecture and subsequent immune responses, with a focus on macrophages. As examples of fibroblast-derived ECM proteins, we examine Collagen Triple Helix Repeat Containing 1 (CTHRC1) and Transforming Growth Factor-β-inducible protein (TGFBI), also known as BIGH3. We address the need for investigation into how diverse fibroblast populations coordinate immune responses by modulating ECM, including the fibroblast-ECM-immune axis and the precise molecular mediators and pathways which regulate these processes. Finally, we will outline how novel research identifying key regulators of ECM deposition is critical for therapeutic development for fibrotic diseases and cancer.

## Introduction

***Fibroblasts orchestrate ECM deposition in steady state and disease:*** Fibroblasts are non-hematopoietic mesenchymal stromal cells which are found in all tissues, regulate tissue structure, and influence the phenotype and localization of tissue resident cell types. ^1^^–^^3^ As a result, fibroblasts play a critical role in tissue homeostasis and tissue repair. Fibroblasts accomplish these functions in part by producing and secreting extracellular matrix (ECM) proteins, which alter the structure and composition of the composite tissue ECM. ^3^ Here, the ECM refers to the diverse network of proteins that generates the three-dimensional structure of tissues. ^4^^–^^6^ In addition, fibroblasts secrete a variety of molecules, including cytokines and chemokines, which can bind the ECM to influence immune cell function and recruitment. ^2^^,^^3^^,^^7^

Under homeostatic conditions, fibroblast-derived ECM proteins provide essential support to resident cells and tissues. However, dysregulation of the ECM can contribute to pathogenic outcomes, including fibrosis. When tissues are injured, local tissue fibroblasts become activated and increase their contractility, secretion of inflammatory mediators, and synthesis of ECM components. ^3^^,^^8^ These changes initiate the wound healing response. ^1^ When damage is limited and non-repetitive, wound healing is efficient, a transient increase in the deposition of ECM components transpires to facilitate the restoration of functional tissue architecture. However, when injury is repetitive or severe such as in chronic inflammatory diseases, ECM components continue to accumulate, which can lead to structural alterations of the ECM, disruption of tissue architecture, organ dysfunction, and ultimately, organ failure. ^1^^,^^4^ In these situations, fibroblasts produce excessive ECM, and changes to the fibrillar collagen network result in pathological tissue stiffness, loss of mechanical compliance, and loss of tissue function. ^2^ As a result, fibrotic disease affects almost all organ systems and is a contributing factor in 45% of all deaths in high income countries. ^8^^,^^9^ Therefore, an appreciation of the interconnectivity between fibroblasts, the ECM, and immune cells is required to understand the causality of fibrotic disease. ^3^ Here we integrate the current understanding of the fibroblast-ECM-immune axis and review the evidence supporting current models defining fibroblast-immune interactions. These studies serve as the basis for future exploration into immune cell regulation by fibroblast-derived ECM molecules.

***ECM form & function:*** The ECM is a dynamic 3-dimensional network of more than 300 different core proteins and matrix-modifying enzymes (often referred to as the matrisome). ^5^^,^^6^^,^^10^ These components are in part produced and assembled by fibroblasts and may either be long lived or transient, making ECM dynamic and sensitive to local and systemic changes. ^2^ Components of the ECM include collagens, proteoglycans (PGs), glycosaminoglycans (GAGs), elastin and elastic fibers, laminins, fibronectin, mucus and other proteins and glycoproteins, such as matricellular proteins. ^2^^,^^4^^,^^5^ The diversity of proteins in these functional groups is immense. For example, there are approximately 28 types of collagens, molecules which provide structural support to tissues. These components may also combine for additional diversity. For example, fibril-forming collagens have abilities to form covalent cross-links and create matrix structures and include collagens I, II, III, V, and XI. These ECM components have a variety of disparate functions while providing architectural support to tissues. For example, collagens provide tissue strength and resilience, PGs form hydrated gels which cushion tissues, and mucus protects barrier surfaces. ^2^^,^^4^ In addition, these components contribute to fundamental processes for tissue development, including cell proliferation, survival, migration, differentiation, autophagy, and angiogenesis. ^4^ These concepts have been reviewed extensively elsewhere. ^4^

There are a variety of other fibroblast-derived molecules and enzymes which modify ECM structure and regulate their degradation, such as matrix metalloproteinases (MMPs). ^1^ Moreover, fibroblasts produce molecules which bind ECM components to alter the composition and function of ECM-resident cells. For example, fibroblasts produce and secrete cytokines, chemokines, and growth factors, which are an implicit part of immune cell regulation. ^2^ It is also notable that immune cells are often connected to the unique matrix surrounding them, and every cell to varying degrees is coated in a glycocalyx, a complex network of sugar rich molecules either free or bound to proteins and/or lipids. ^2^^,^^11^ As a result, the ECM regulates the signaling, functions, properties, and morphology of residing immune cells in addition to providing structural support for tissues. ^4^ These properties may be altered in a tissue-specific manner. ^3^ Specific ECM phenotypes configure the different tissues, including epithelial, muscle, connective, and more to meet the requirements for optimal tissue function. ^4^ Here, the prevalence of different, tissue-specific heterogeneous fibroblast subsets with unique gene expression signatures can further alter the ECM and therefore tissue characteristics. ^12^ Finally, fibroblasts have unique functions depending on disease states. Fibroblasts that become activated due to repeated injury and chronic inflammation are typically referred to as myofibroblasts and have altered gene expression profiles that drive fibrotic disease. Below, we highlight critical immune-facing functions of fibroblast- and myofibroblast-derived ECM components in steady-state and disease. ^5^

### ECM regulations of fibroblast-macrophage interactions

**Figure 1.**
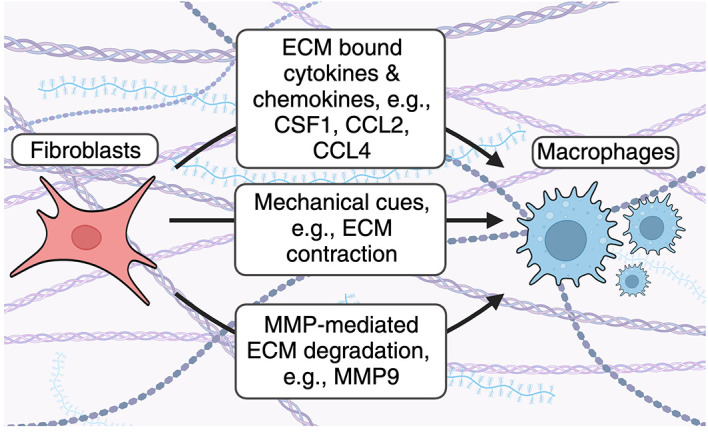
Fibroblast influence macrophage accumulation by altering the composition of the ECM. Fibroblasts use several mechanisms to recruit macrophages to the ECM. Fibroblast-derived cytokines and chemokines bind the ECM and enhance accumulation of ECM-associated macrophage. Fibroblast contraction remodels collagen fibers to enhance macrophage recruitment. Fibroblasts secrete MMPs to cleave ECM- and ECM bound proteins to enhance macrophage accumulation in the ECM.

***Fibroblast derived cytokines and chemokines decorate the ECM to influence fibroblast-macrophage interactions.*** Fibroblasts produce cytokines and chemokines which bind the ECM and are critical for immune cell recruitment and function in steady-state and disease ^2^^,^^13^ ( Figure 1). For example, multiple *in vitro* and tissue-restricted *in vivo* studies have demonstrated that a relationship exists between fibroblasts and macrophages, an innate immune cell which orchestrates long-lived adaptive immune responses through phagocytosis, antigen presentation, and immunological mediator secretion. ^13^ Fibroblasts and macrophages are found in almost all tissues and multiple studies indicate that fibroblast-derived CSF1, which can decorate the ECM, is a critical factor for macrophage survival. Using *in vitro* studies, Zhou et al. demonstrated that macrophages and fibroblasts form stable cell circuits that are resistant to perturbations and that cell-to-cell contact increased cell circuit survival because of local exchange of growth factors, including CSF1. ^14^ In addition, *in vivo* studies have demonstrated that fibroblastic reticular cells (FRC) are required to establish the lymph node (LN) macrophage niche. D’Rozario et al. demonstrated that genetic ablation of FRC using a genetic tool in which Chemokine (C-C motif) ligand 19 ( *Ccl19*)-expressing cells could be depleted using diphtheria toxin resulted in rapid loss of monocytes and macrophages from LN in two separate *in vivo* models. ^15^ Moreover, single-cell RNA sequencing (scRNA-seq) of murine brachial lymph nodes revealed that FRC subsets broadly expressed master macrophage regulator CSF1 and functional assays containing purified FRC and monocytes showed that CSF1R signaling was sufficient for macrophage survival. ^15^ Comparative analysis demonstrated these effects were conserved between mice and humans. ^15^

Monocytes, which can give rise to macrophages, have also been shown to require stromal cell derived CSF1. Emoto et al. demonstrated that CSF1 produced by sinusoidal endothelial cells was required for the survival of Ly6C- monocytes. Conversely, CSF1 produced by both endothelial and *Lepr*+ perivascular stromal cells were required for the survival of Ly6C ^hi^ monocytes. ^16^ Taken together, these studies demonstrated that macrophage survival in specific tissues is regulated by stromal-derived CSF1.

As different tissue-specific fibroblast subsets can produce different levels of CSF1, ^17^ additional studies are required to delineate which subsets are critical for macrophage homeostasis. Moreover, the functional role of macrophages sustained by fibroblast-derived CSF1 in disease requires further study. Previous work by Zhu et al. has demonstrated that a pharmacologically induced CSF1 blockade in mouse pancreatic tumour models results in increased antigen presentation and productive antitumour T cell responses. ^18^ Despite this, the precise contribution of fibroblast-derived CSF1 in antitumour immunity is not well understood. Furthermore, the functional contributions of these macrophages in chronic inflammation and cancer also require subsequent study.

In addition to cytokines, activated fibroblasts, including myofibroblasts, produce ECM-binding macrophage chemoattractants indicating that fibroblasts are capable of recruiting monocytes and macrophages during injury, infection, and inflammation. ^19^^,^^20^ For example, the bacterial ligand lipopolysaccharide (LPS), which activates toll-like receptor (TLR)4, has been shown to enhance expression of macrophage chemoattractants *Ccl2* and *Ccl4* in myofibroblast progenitor cells in a mouse model of liver fibrosis. ^19^ As LPS-mediated TLR4 activation occurs widely in anti-bacterial immunity, this suggests that fibroblasts can respond to pathogen-associated molecular patterns (PAMP) to recruit immune cell subsets, including macrophages, in a variety of anti-infective contexts. However, the role of fibroblast-mediated macrophage recruitment in additional anti-infective immune responses, as well as chronic inflammation, requires future investigation.

***Fibroblasts influence macrophage migration through mechanical cues.*** Previous studies have also demonstrated that fibroblasts can influence macrophage dynamics by regulating the physical mechanics of the ECM ( Figure 1). Pakshir and colleagues showed that contractile myofibroblasts drive mechanical cues through local remodeling of collagen fibers resulting in increased macrophage migration directly towards myofibroblasts using 3D collagen gels *in vitro.* ^21^ Migration occurred independently of chemotaxis and required macrophage-mediated attachment to collagen via the α2β1 integrin and stretch-sensitive ion channels. ^21^ Similar observations have been described in several fibroblast-like cells, ^22^^–^^24^ indicating the importance of these biomechanical mechanisms for cell communication and movement across tissues.

***Fibroblast-derived matrix metalloproteinases degrade ECM proteins to enhance macrophage recruitment.*** Matrix metalloproteinases (MMPs) are a family of enzymes which degrade ECM proteins. These enzymes are either secreted or attached to cell surfaces, confining their activity to membranes, secreted proteins, or proteins within the extracellular space. ^25^ MMPs have a complex role in regulating inflammation by acting on a multitude of immunological mediators, including antimicrobial host defense peptides, cytokines, chemokines, and ECM proteins as reviewed in detail here. ^25^ In addition, matrix metalloproteinases are enhanced in stromal cells, including fibroblasts, in response to pro-inflammatory cytokines. ^26^^,^^27^ As a result, modulation of the ECM during inflammation alters immune cell migration and ECM resident cell types, including macrophages ( Figure 1). For example, Shubayev et al. demonstrated that TNF-α-mediated MMP9 (also known as gelatinase B) production enhances macrophage recruitment in a model of peripheral nerve injury. ^28^ Gong et al. demonstrated that mice deficient for the ECM-bound protease plasminogen (Plg) had decreased trans-ECM macrophage migration and decreased MMP9 activation. ^29^ In addition, the authors demonstrated that MMP9 administration to Plg deficient mice resulted in increased macrophage accumulation in the ECM, ^29^ suggesting that Plg activates MMP9 to increase ECM resident macrophages. Moreover, Tan et al. demonstrated that MMP9 can also cleave the ECM protein osteopontin (OPN) to enhance macrophage recruitment in a mouse model of renal fibrosis. ^30^ Despite this, additional studies are required to understand how MMP-mediated degradation of other ECM components alters immune cell recruitment and fibrotic disease development.

## Fibroblast heterogeneity dictates ECM deposition

**Figure 2.**
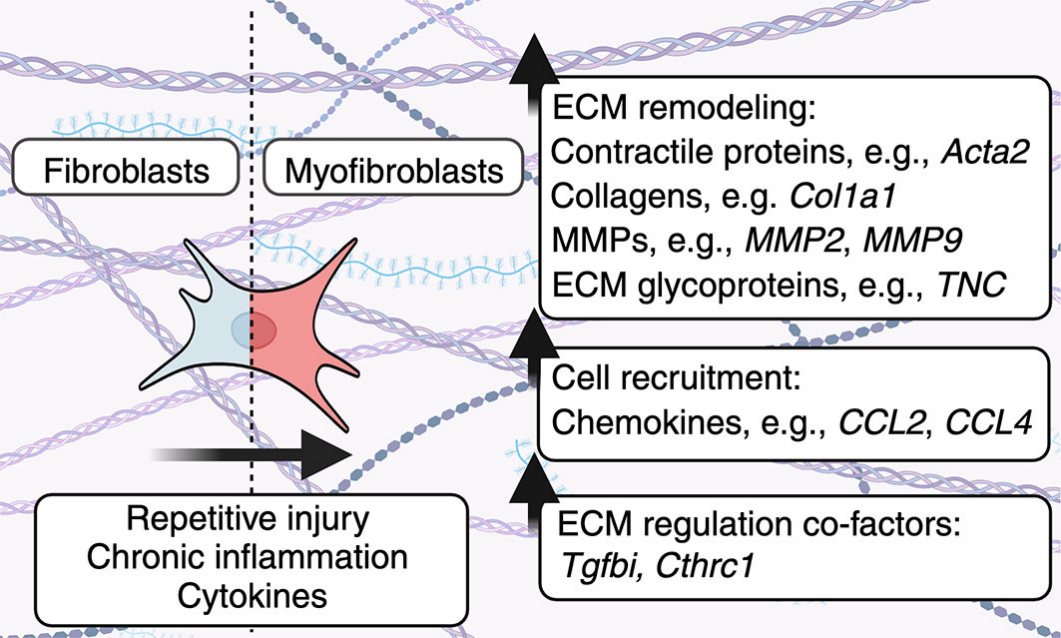
Heterogeneous fibroblast subsets alter the ECM. Fibroblasts become activated in response to repetitive injury, chronic inflammation or can be activated by cytokines to enhance the production of ECM components. As a result, heterogeneous fibroblast subsets can alter the physical properties of the ECM, as well as ECM-resident cells. In addition, fibroblasts produce molecules including TGFBI/BIGH3 and CTHRC1 which play multifaceted roles in regulating fibroblast-mediated ECM deposition.

In addition to fibroblast-derived components, immune-derived cytokines and growth factors shape play a critical role in fibrosis and fibrotic diseases by altering fibroblast heterogeneity and therefore ECM deposition. Below, we highlight the relationship between inflammation, fibroblast heterogeneity, and ECM deposition ( Figure 2). Moreover, we outline the role that modulators of growth factor signaling play in shaping ECM deposition and subsequent fibrotic disease.

***Immunological and inflammatory cues dictate fibroblast heterogeneity and ECM deposition.*** ECM composition is regulated by diverse fibroblast subsets in health and disease. Here, pro-inflammatory cytokines, growth factors, and other ECM proteins can convert fibroblasts into myofibroblasts. ^31^ Relative to fibroblasts found in unperturbed tissues, myofibroblasts exhibit increased expression of contractile protein genes, such as alpha smooth muscle actin ( *Acta2*) ^32^ as well as other of ECM proteins. ^33^ For example, fibrotic cells are heterogeneous and distinct between uninjured and injured skin. Building on previous experiments in mice which demonstrated that embryonic mesenchymal precursors expressing *Engrailed* ( *En1*) or *Delta-like homolog* 1 ( *Dlk/Pref1*) generate skin fibroblast and adipocyte lineages, Shook et al. demonstrated that the predominant population of ECM-producing myofibroblasts in the skin are adipocyte precursor cells (AP) derived from *En1*-lineage traced fibroblasts. ^34^ In addition, this study demonstrated that wound bed myofibroblasts upregulated *Acta2* and *Col1a1* mRNA expression compared to fibroblast populations from uninjured skin. ^34^ Further, the authors compared the transcriptional profiles of 2 major populations of fibrotic mesenchymal cells that are enriched in skin wound beds: APs and CD29 ^high^ cells. APs had greater expression of ECM components/regulators including collagens *Col5a2*, *Col14a1*, as well as matrix metalloproteinases *Mmp2*, *Mmp3*, *Mmp23*, *Mmp27.* Conversely, CD29 ^high^ fibroblasts expressed elevated levels of *Col6a3*, *Col7a*, *Mmp13*, and *Tnc.* Moreover, platelet-derived growth factor C ( *pdgfc*) secreted by CD301b-expressing macrophage triggered the proliferation of APs, but not other myofibroblasts, highlighting the role of immune-derived growth factors in shaping fibroblast heterogeneity.

Transforming growth factor beta (TGF-β) is another major immune-derived growth factor which drives the expression of myofibroblast-associated genes. TGF-β is produced by a variety of cells, including macrophages, under inflammatory conditions and drives myofibroblast activation. ^13^ In the canonical TGF-β pathway, TGF-β targets the TGF-β receptor complex in cells to drive the activation of intracellular kinases, including SMAD2/3, co-SMAD, and SMAD4 to upregulate ECM and myofibroblast-associated genes, such as C *ol1a1* and *Acta2.* ^35^^,^^36^ In addition, TGF-β can also signal through non-canonical signaling pathways and activate all three mitogen-activated protein kinase (MAPK) pathways and proteins, including extracellular signal-regulated kinase (ERK), p38 MAPK, and c-Jun-N-terminal kinase (JNK). ^35^ TGF-β-mediated signaling through these pathways may occur in either a Smad-dependent or -independent fashion. ^35^ TGF-β also activates Rho GTPase signaling and the PI3 kinase/Akt pathway. ^35^ As TGF-β is crucial for myofibroblast development and ECM production, modulators of TGF-β-signaling may be co-factors that affect myofibroblast phenotypes and ECM production from these cells. We define co-factors as proteins that are required for optimal signaling of another protein, such as TGF-β.

***Fibroblast-derived CTHRC1 as a regulator of ECM deposition.*** Collagen triple helix repeat containing 1 ( *CTHRC1)*, a secreted ECM protein, has recently been identified as a critical modulator of ECM protein modulation and wound healing. ^37^^,^^38^ Human CTHRC1 contains a N-terminal 30 amino acid hydrophobic signal peptide secretory domain, a short collagen triple helix repeat (CTHR) domain consisting of 12 repeats of the Gly-X-Y motif, and a highly conserved C-terminal domain with similar structure of the globular C1q domain of Collagen VIII. ^39^^,^^40^ *CTHRC1* is expressed in macrophages, myofibroblasts, endothelial cells as well as mesenchymal-derived cells ^41^ and as a result, *CTHRC1* expression occurs in multiple tissues, including the bone, ^42^ the lung, ^43^ and tumours. ^44^

**Figure 3.**
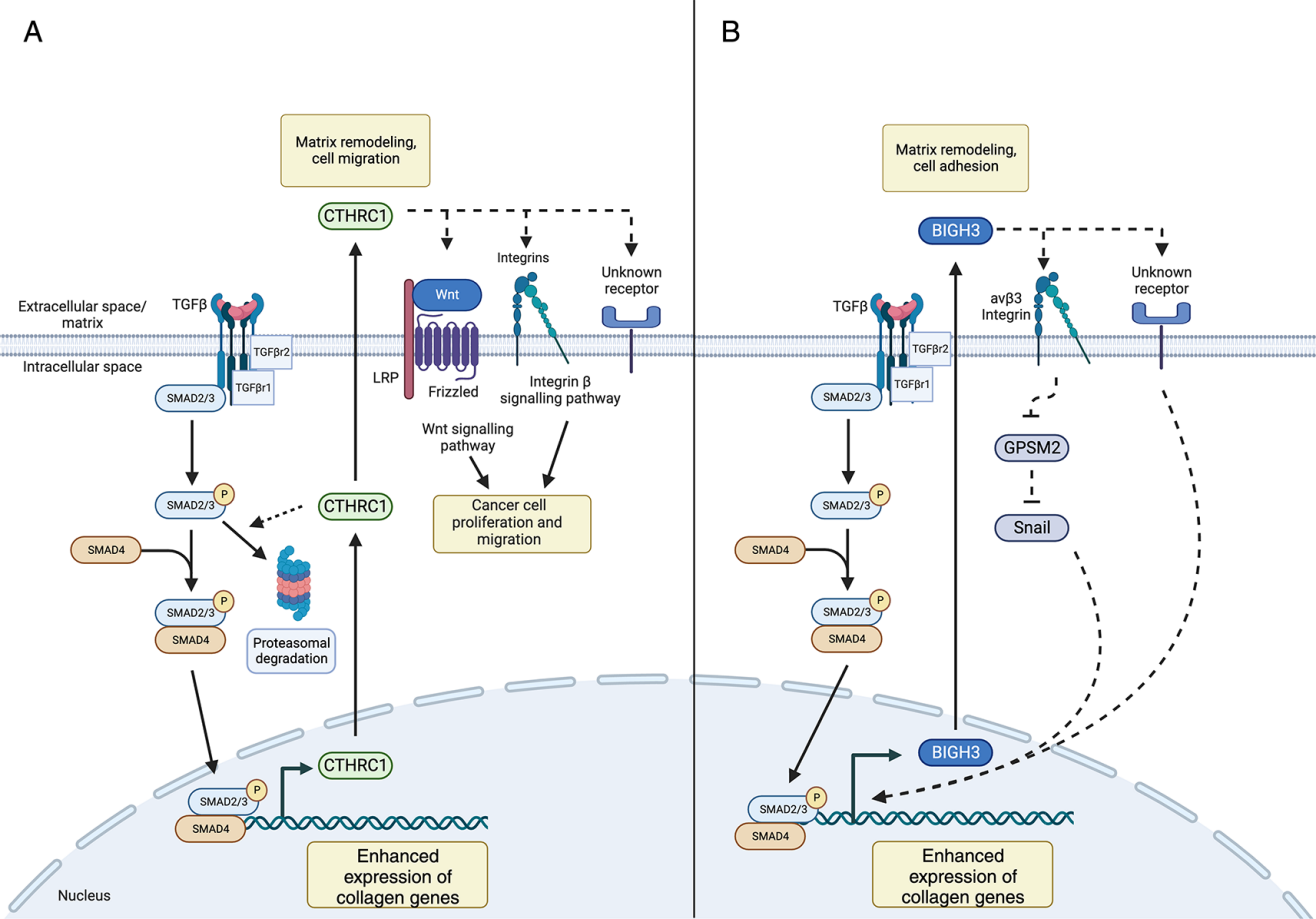
Proposed mechanisms of CTHRC1- and BIGH3-mediated ECM modulation. (A) TGF-β signaling enhances production of extracellular matrix proteins, as well as CTRHC1 *,* resulting in negative feedback loop which may limit its own production. Secreted CTHRC1 can bind to collagens and integrins and has been implicated in cancer cell proliferation and migration by influencing Wnt and integrin β signaling pathways. (B) TGF-β signaling enhances BIGH3 production which subsequently binds collagens and integrins. Proposed pathways of BIGH3-mediated collagen production include inactivation of the GPSM2 signaling pathway and activation of Snail, a transcription factor which influences collagen production. Legend: dashed lines represent prospective/predicted interactions.

Previous studies suggest that CTHRC1 modulates TGF-β signaling ( Figure 3A). For example, *Cthrc1* modulates TGF-β signaling by promoting the degradation of canonical signaling intermediates, including Smad2/3, and has been suggested to influence Wnt and β-integrin signaling to affect cancer cell proliferation and metastasis. ^41^ Guo et al. showed that *Cthrc1* modulated β-integrin signaling by enhancing phosphorylation of Focal adhesion kinase (FAK) to promote metastasis of epithelial ovarian cancer (EOC) cells. ^45^ In fibroblast and smooth muscle cells, CTHRC1 levels are associated with increased cell migration *in vitro.* ^46^ Other reports suggest that CTHRC1 may negatively regulate TGF-β-mediated signaling pathways. For example, Leclair et al. demonstrated that overexpression of *CTHRC1* in smooth muscle cells reduced levels of phosphorylated Smad2/3, which is required for TGF-β-mediated gene expression and collagen production. ^47^ As *Cthrc1* expression is enhanced in response to TGF-β *in vitro,* ^46^ mechanistic evidence suggests that TGF-β and CTHRC1 may act in a negative-feedback loop to limit TGF-β-induced ECM deposition. Due to conflicting reports on the role of CTHRC1 in a disease context, additional studies are required. Future queries delineating the precise relationship between CTHRC1 in TGF-β-mediated signaling and the role of CTHRC1 in a disease context must focus on cell- and tissue-specific mechanisms.

CTHRC1 has been shown to mark lung myofibroblasts in the context of models of lung fibrosis in murine systems and idiopathic pulmonary fibrosis (IPF) in humans. ^17^^,^^43^^,^^48^^–^^50^ *Cthrc1* expression may also distinguish two disease-associated myofibroblast clusters across tissues. ^17^ One disease-associated myofibroblast cluster is marked by the simultaneous expression of *Lrrc15* (leucine-rich repeat 15) and *Cthrc1* expression, whereas the other is only marked by *Cthrc1* and not *Lrrc15* expression. ^17^ scRNA-seq-based characterization of collagen-producing cells in fibrotic mouse lungs demonstrated that the *Cthrc1+* fibroblast cluster expresses the highest levels of ECM proteins, including *Col1a1* and *Col3a1.* ^43^

Tsukui et al. used transgenic mice to track and ablate *Cthrc1*-expressing fibroblasts in lung fibrosis. Lineage tracing using Ctrhrc1-CreER mice crossed to Rosa26-tdTomato mice demonstrated that *Cthrc1*-expressing lung fibroblasts expand in a mouse model of bleomycin-induced pulmonary fibrosis. *Cthrc1*-Cre-ER-labeled cells showed elevated expression of ECM genes, including *Col1a1* and *Postn* (periostin). Further, depletion of *Cthrc1*-expressing lung fibroblasts using Ctrhrc1-CreER mice crossed to Rosa26-tdTomato and Diphtheria toxin receptor expressing mice attenuated hydroxyproline, a marker of collagen deposition following administration of bleomycin. ^49^ Taken together, these results suggest that *Cthrc1* expressing cells drive ECM deposition and may promote fibrosis.

Conversely, different studies have demonstrated that CTHRC1 may limit ECM deposition and fibrosis. Global *Cthrc1* knock-out (KO) resulted in increased hydroxyproline levels and tissue remodeling in the lungs of mice following bleomycin administration. ^48^ In a mouse model of vascular fibrosis, *Cthrc1* was transiently expressed by adventitial fibroblasts following injury and was associated with decreased collagen deposition in cross-sections of carotid arteries. ^46^ The discrepancies between these studies may highlight the cell-, tissue-, and disease-specific role of *Cthrc1.* As such, further studies are needed to determine the precise role of CTHRC1 in diverse fibrotic diseases. In addition, specific genetic tools, including fibroblast-specific *Cthrc1* KO mice or fluorescently-labelled *Cthrc1*-expressing cells are required to properly assess the pathogenicity of *Cthrc1+* myofibroblasts in different tissues and disease models.

***Fibroblast- and macrophage-derived BIGH3 is associated with increased collagen deposition.*** Transforming growth factor beta inducible protein (TGFBI), is also known as keratoepithelian, ^51^ and is referred to here as beta-inducible growth hormone 3 (BIGH3) is produced by fibroblasts and macrophages. ^52^ BIGH3 was discovered in 1992 as a gene highly upregulated by A594 lung adenocarcinoma cells following TGF-β stimulation. ^53^ The structure of BIGH3 has not been solved; however, early characterization of this protein using cDNA sequence analysis suggested that it contains a secretory signal peptide, four fasciclin 1 (FAS1) domains that mediate binding to extracellular matrix proteins, such as collagens, and a RGD motif that enables the binding of this protein to integrin receptors. ^53^^–^^55^ *BigH3* has been shown to be expressed by fibroblasts upon TGF-β exposure ^56^ and by macrophages in ‘M2’ polarization conditions *in vitro.* ^57^^–^^59^ In monocytes, *BigH3* is produced following ingestion of apoptotic cells. ^52^ Upon secretion by fibroblasts and macrophages, BIGH3 binds ECM components, such as collagens, ^60^ and subsequently regulates the α _v_β _5_-dependent adhesion and migration of mesenchymal and ectodermal-lineage cells, including fibroblasts, chrondrocytes, osteoblasts, keratinocytes, and endothelial cells. ^58^^,^^59^^,^^61^

This gene is also a paralog of periostin (POSTN). ^62^ Studies investigating the role of BIGH3 in disease indicated tissue-specific functions. Schwanekamp et al. demonstrated that *BigH3* was enhanced in response to myocardial infarction in the heart. Despite this, cardiac-specific deletion of *BigH3* did not alter cardiac disease or fibrosis post-myocardial infarction. ^63^ In the colon, TGF-β signalling plays a major role in promoting fibrotic lesions in inflammatory bowel diseases (IBD). ^36^ Aligned with this *BigH3*, as well as ECM genes *COL1A1*, *COL1A2,* and *COL3A1* are upregulated in patients with fibrotic IBD, suggesting that combined BIGH3 and TGF-β signalling may accelerate colon fibrosis. ^36^^,^^64^ In the lungs, Ahlfeld et al. demonstrated that *BigH3* is elevated in response to lung injury ^63^ and *BigH3* KO mice at early ages had documented lung developmental abnormalities, lack of elastin-positive tips, reduced proliferation, and abnormally persistent *aSMA* myofibroblasts, which resolve by adulthood. ^65^ The authors also demonstrated that lungs in *BigH3* deficient mice had reduced elastic recoil and gas exchange efficiency. ^65^ Zhang et al. demonstrated that *BigH3* deficient mice had stunted growth *in vivo,* as well as enhanced proliferation and *cyclin* D1 expression *ex vivo*, suggesting that BIGH3 may limit cellular proliferation. ^66^ It remains to be seen if adult animals exhibit these defects or compensatory effects mask phenotypes seen in younger animals.

In addition to impacting lung development, *in vitro* studies have demonstrated that BIGH3 can promote collagen deposition in lung fibroblasts. ^67^ Yang et al. found that siRNA-mediated knockdown (KD) of *BigH3* in human lung fibroblast cultures resulted in decreased TGF-β- collagen 1 (Col 1) and αSMA production, demonstrating that BIGH3 is necessary to produce these ECM proteins. ^67^ The molecular mechanism by which BIGH3 mediates *Col1a1* deposition downstream of TGF-β is unclear. It has been posited based upon *in vitro* studies using lung fibroblast cell lines that BIGH3 mediates its effects via a multi-part mechanism. Briefly, TGF-β first elicits BIGH3 expression, then BIGH3 binds a multitude of integrin receptors, including α _1_β _1,_^68^ α _3_β _1,_^69^^,^^70^ α _v_β _3_^58^ or α _v_β _5_^58^ integrin. BigH3 then drives activation of PI3K-signaling ^70^ and increased Snail expression, encoded by *Snail1*, via downregulation of a Snail negative regulator G-protein signaling modulator 2 (GPSM2). ^58^^,^^67^ Aligned with this, Nacu et al. demonstrated that lung fibroblasts stimulated with BIGH3 had increased Collagen 1 protein abundance. ^52^ Taken together, these studies revealed that BIGH3 is both necessary and sufficient to induce collagen production in lung fibroblasts. However, further study is required to characterize the full scope of ECM deposition changes in response to BIGH3.

Mechanistic studies indicate that BIGH3 modulates TGF-β-mediated signal transduction pathways ( Figure 3B). In addition to studies defining the tissue-specific nature of BIGH3 and the mechanisms of its effect on ECM deposition, further investigation is required into additional roles of BIGH3 in the context of fibroblasts and macrophage spatiality, including mechanical sensing, and/or monocyte/macrophage chemotaxis. As such, cell- and tissue-restricted BIGH3 KO models paired with high-resolution techniques, such as scRNA-seq are required to examine the role that BIGH3 plays in modulating ECM deposition, development, and disease.

## Conclusions

**Figure 4.**
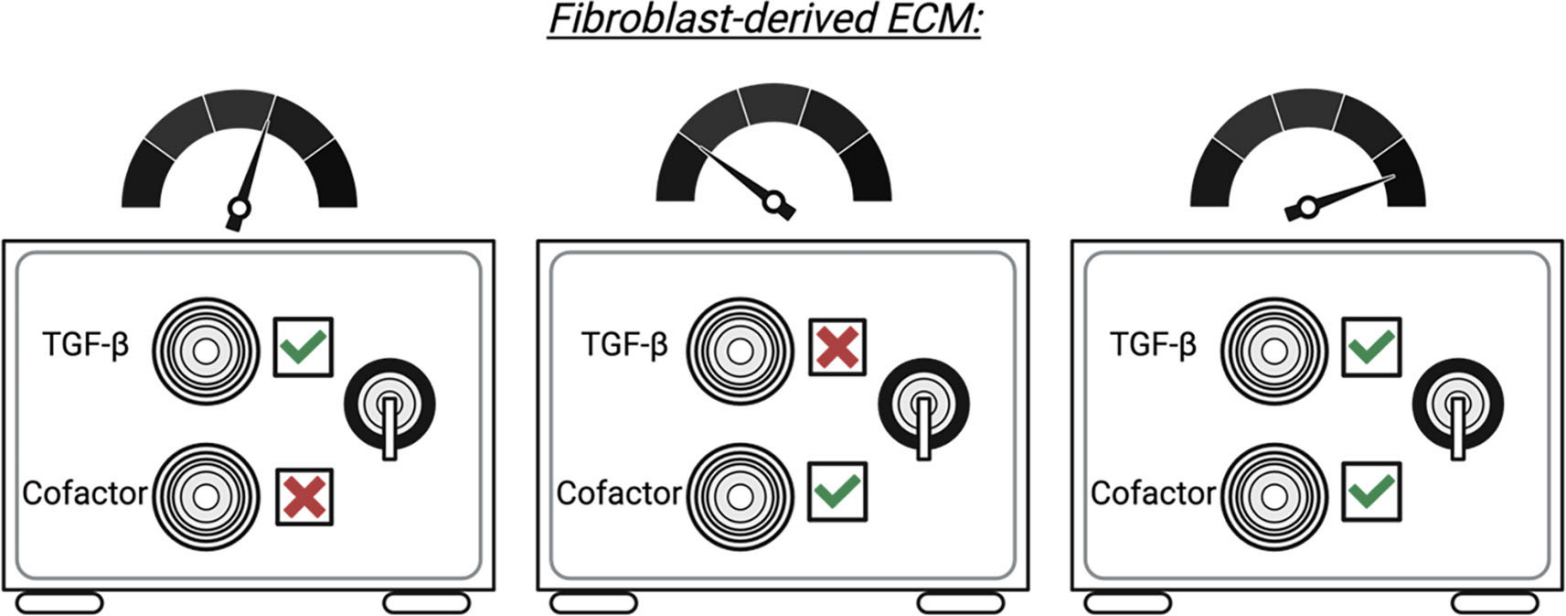
Proposed model for cofactor-mediated ECM deposition in fibroblasts. The role, mechanism, and source of cofactors, including CTHRC1 or BigH3 in health and fibrotic disease are not well defined. Further studies are required to determine key aspects of BIGH3 and CTHRC1 biology and how this drives ECM deposition and fibrotic disease in combination with other pro-fibrotic mediators.

The interface between fibroblasts, the immune system, and the ECM is wide and complex. As a result, an appreciation that fibroblasts and the immune system act in a finely tuned and integrated system for ECM deposition is required for understanding the biology of fibroblasts, as well as their roles in ECM deposition and fibrotic disease. Current research focus on the relationship between fibroblasts and ma crophages. As a result, additional studies are required to determine whether the relationships between fibroblasts and other immune cell subsets can shape ECM deposition or if macrophages are required as an intermediate. Despite this, targeting the fibroblast-ECM-immune axis may provide opportunities to prevent fibrotic disease. For example, defining the mechanism of action of immune-derived ECM modulators, including CTHRC1 and BIGH3, may provide an opportunity to modulate disease-associated myofibroblast development and/or prevent excessive fibrosis. Indeed, an attractive approach to ameliorate TGF-β related fibrosis may be to target potential co-factors or effect modulators within the TGF-β signaling pathway, such as CTHRC1 or BIGH3. It is possible that fibrotic deposition by fibroblasts is akin to opening a safe that requires two keys: one representing TGF-β and the other representing an essential co-factor, such as CTHRC1 or BIGH3 ( Figure 4). To this end, we envision that intervention strategies specifically targeting TGF-β signalling cofactors (or the myofibroblasts that produce them) may limit fibrotic disease and prevent off-target effects that are common among current therapies.

## Author contributions

Writing – Anthony Altieri, Grace Victoria Visser, Matthew B Buechler.

## Data Availability

No data are associated with this article.
