# Supplementary material for: Enter the Matrix: Fibroblast-immune cell interactions shape extracellular matrix deposition in health and disease
Source: F1000Res. 2024 Dec 5;13:119. Originally published 2024 Feb 19. [Version 2] doi: 10.12688/f1000research.143506.2 (PMC11781523; doi:10.12688/f1000research.143506.2)
Supplement: Peer review history

# Reviewer report: Reviewer response for version 2

**DOI:** 10.5256/f1000research.173782.r347955

**Published:** 2025-01-11

**Authors:** George Kollias

**Affiliations:** 1 Biomedical Sciences Research Center “Alexander Fleming”, Vari, Greece

In the current review, Altieri *et al*. revisit the literature to highlight the ECM signals that mediate the fibroblast-macrophage interactions in the context of wound healing, fibrosis and cancer. They additionally summarize the current knowledge on the matrisome proteins CTHRC1 and TGFBI (BIGH3), and discuss their impact on ECM accumulation in fibrotic and malignant tissues. Overall, the manuscript is well-written and the authors comprehensively present the diverse nature of ECM signals (mechanical cues mediated by core matrisome proteins such as collagens, and biochemical signals mediated by matrisome-associated proteins, such as secreted factors (e.g. CSF1) and ECM regulators (MMP9)). Nonetheless, some major concerns need to be remarked:

 1) The review’s title and abstract predispose the reader for a collection of studies that focus on the fibroblast-ECM-macrophage axis in fibrosis and cancer. Nevertheless, the manuscript's content does not align with its title, since the sender and/or receiver cell of the ECM signal is undefined in most of the references included. For example, in Figure 1 the authors schematically claim that fibroblast-secreted MMP9 promotes the accumulation of macrophages in the tissues. The respective studies by Shubayev *et al*., Gong *et al*., and Tan *et al*. indeed support the macrophage-recruiting role of MMP9, but they do not define the fibroblast as the cellular source of the enzyme. In the paper by Tan *et al*. in particular, the authors describe the tubular epithelial cells (TEC) as the major MMP9 producer. Consequently, Figure 1 should be modified accordingly to avoid misinterpretations.

 Additional ambiguity is detected regarding CTHRC1 and TGFBI. In the abstract, these molecules are defined as ‘fibroblast-derived ECM proteins’, whereas in the main text they are described as ‘immune-derived co-factor candidates’. Moreover, it does not become clear to the reader how the discussion on these proteins serves the purpose of the manuscript, given that no crosstalk between fibroblasts and macrophages through CTHRC1/TGFBI is reported.

 Overall, we recommend a cautious reconstruction of the text across the manuscript in a way that the references will reliably reflect the scope of the review.

 2) Since fibroblast-macrophage interactions in fibrosis are in the spotlight of the review, the authors should consider dedicating a section to discuss the emerging topic of scar-associated macrophages (SAMacs)  and how their communication with pro-fibrotic fibroblasts affects the disease course across tissues. This addition will be of particular relevance to the review’s main focus, as various ligands that have been described to mediate the crosstalk of the two fibrotic cell types constitute ECM-associated components (SPP1, IL-1β, PDGF), and are currently trending as potential antifibrotic therapeutic targets ^1–6^.

*3) Fibroblasts orchestrate ECM deposition in steady state and disease: *In addition to the pathological consequences of aberrant fibroblast activation upon injury, it is worth mentioning the reviews by Koliaraki *et al*. (JEM, 2020 and Nature Immunology, 2020) ^7,8^ to also discuss the immunomodulatory effects of CAFs and ECM in the context of cancer.

Is the review written in accessible language?

Yes

Are all factual statements correct and adequately supported by citations?

Partly

Are the conclusions drawn appropriate in the context of the current research literature?

Partly

Is the topic of the review discussed comprehensively in the context of the current literature?

Partly

Reviewer Expertise:

Inflammatory diseases, Intestinal fibrosis, Colon cancer

I confirm that I have read this submission and believe that I have an appropriate level of expertise to confirm that it is of an acceptable scientific standard, however I have significant reservations, as outlined above.

## References

[1] : Resolving the fibrotic niche of human liver cirrhosis at single-cell level. *Nature* .2019;575(7783) : 10.1038/s41586-019-1631-3 512-518 10.1038/s41586-019-1631-3 31597160 PMC6876711

[2] : Platelet-instructed SPP1+ macrophages drive myofibroblast activation in fibrosis in a CXCL4-dependent manner. *Cell Rep* .2023;42(2) : 10.1016/j.celrep.2023.112131 112131 10.1016/j.celrep.2023.112131 36807143 PMC9992450

[3] : Targeting immune-fibroblast cell communication in heart failure. *Nature* .2024;635(8038) : 10.1038/s41586-024-08008-5 423-433 10.1038/s41586-024-08008-5 39443792 PMC12334188

[4] : Chromatin remodelling drives immune cell-fibroblast communication in heart failure. *Nature* .2024;635(8038) : 10.1038/s41586-024-08085-6 434-443 10.1038/s41586-024-08085-6 39443808 PMC11698514

[5] : Spatial multi-omic map of human myocardial infarction. *Nature* .2022;608(7924) : 10.1038/s41586-022-05060-x 766-777 10.1038/s41586-022-05060-x 35948637 PMC9364862

[6] : Identification of a broadly fibrogenic macrophage subset induced by type 3 inflammation. *Sci Immunol* .2023;8(82) : 10.1126/sciimmunol.add8945 eadd8945 10.1126/sciimmunol.add8945 37027478

[7] : Unfolding innate mechanisms in the cancer microenvironment: The emerging role of the mesenchyme. *J Exp Med* .2020;217(4) : 10.1084/jem.20190457 10.1084/jem.20190457 32044979 PMC7144533

[8] : The mesenchymal context in inflammation, immunity and cancer. *Nat Immunol* .2020;21(9) : 10.1038/s41590-020-0741-2 974-982 10.1038/s41590-020-0741-2 32747813

# Reviewer report: Reviewer response for version 2

**DOI:** 10.5256/f1000research.173782.r347953

**Published:** 2025-01-07

**Authors:** Spyridon Makris

**Affiliations:** 1 University College London, London, UK

This review provides valuable insight on the complex interactions between fibroblasts and macrophages in health and disease. The authors elegantly link multiple scRNA-seq, and other transcriptional studies, across multiple tissues to describe the importance of ECM deposition by fibroblast in health and disease. This review is important as it examines the fibroblast:macrophage crosstalk and how it can affect disease outcome. The authors use a large variety of studies to show to show how fibroblasts can alter organ structural properties and regulate the immune responses by secreting ECM and ECM-bound molecules. There is particular focus on ECM-derived proteins, CTHRC1 and TGFBI (BIGH3), as modulators of the ECM proteins. As the authors rightly state, more studies on the crosstalk between fibroblasts and immune cells and how that would affect ECM deposition. Finally, they describe methods by which the TGF-beta signalling cofactors could provide potential therapeutic targets.  

**Minor Points**:

- **Figure 2** not easy to comprehend and in its current form. It would benefit if authors rearranged the figure slightly and provided numbers. The reader should then be able to follow how fibroblast transition toward heterogeneity occurs and how that would link to changes in ECM remodelling, Cell recruitment and ECM regulation of co-factors.
- At end of **ECM form & function** section, letters are missing after IFN-, TNF- and IL-1.
- Figure sizes are not identical and it is difficult to read the small text in Figure 3. I suggest the authors adjust both the font and figure sizes to make it easier for the reader.
- **Figure 3** Merge the initial heading “Matrix remodeling, cell migration, Collagen production”

Is the review written in accessible language?

Yes

Are all factual statements correct and adequately supported by citations?

Yes

Are the conclusions drawn appropriate in the context of the current research literature?

Yes

Is the topic of the review discussed comprehensively in the context of the current literature?

Yes

Reviewer Expertise:

Immunology, Stromal Immunology, Virology, Respiratory infections, Innate immunity

I confirm that I have read this submission and believe that I have an appropriate level of expertise to confirm that it is of an acceptable scientific standard, however I have significant reservations, as outlined above.

# Reviewer report: Reviewer response for version 2

**DOI:** 10.5256/f1000research.173782.r346224

**Published:** 2024-12-06

**Authors:** Valentina Pucino

**Affiliations:** 1 Kennedy Institute of rheumatology, Oxford University, Oxford, UK; 2 Department of Clinical and Experimental Medicine, University of Pisa, Pisa, Tuscany, Italy

The authors answered to all my comments and I have no further comments to make. I am happy for the paper to be indexed in the current version.

Is the review written in accessible language?

Yes

Are all factual statements correct and adequately supported by citations?

Yes

Are the conclusions drawn appropriate in the context of the current research literature?

Partly

Is the topic of the review discussed comprehensively in the context of the current literature?

Partly

Reviewer Expertise:

Immunology, Immunometabolism, fibroblast biology, T cell biology, autoimmunity.

I confirm that I have read this submission and believe that I have an appropriate level of expertise to confirm that it is of an acceptable scientific standard.

# Reviewer report: Reviewer response for version 1

**DOI:** 10.5256/f1000research.157178.r262987

**Published:** 2024-05-17

**Authors:** Valentina Pucino

**Affiliations:** 1 Kennedy Institute of rheumatology, Oxford University, Oxford, UK; 2 Department of Clinical and Experimental Medicine, University of Pisa, Pisa, Tuscany, Italy

I find this review timely and well written. It summarizes the latest findings on fibroblast biology and fibroblast-derived molecules offering an insight on potential therapeutic options to target fibroblast-mediated diseases such as fibrosis where therapeutic treatments are lacking. This is a hot topic at the moment. See detailed report below.

****

 This review from Altieri et al. recapitulates the most recent findings in the field of fibroblasts. In addition, the authors discuss how fibroblasts coordinate immune responses, especially macrophages’ functions, by modulating ECM, and outline the molecular mediators such as CTHRC1 and TGFB and pathways regulating these processes. This is a very well written and timely review.

**Minor points:**

 Add and discuss the following references:

***Fibroblasts orchestrate ECM deposition in steady state and disease***: I would add few more words on fibroblasts heterogeneity here. Some key references below:

 Gauthier V,et.al., 2023 [Ref 1], Korsunsky I, et.al.,2022 [Ref 2], Muhl L, et.al., 2020 [3]

***ECM form & function***:  it’s worth to mention and discuss the paper by Horsnell et al 2023 in addition to reference number 4.

***Fibroblast derived cytokines and chemokines decorate the ECM to influence fibroblast-macrophage interactions***: please add and discuss the following references which focus on fibroblast/macrophage interactions. Ng MTH, et.al.,2024  [Ref 5],Zhou X, et.al.,2022 [Ref 6], Meizlish ML, et.al., 2024 [Ref 7]

 Recent evidence has demonstrated that metabolic intermediates can also fuel stromal cell responses and ECM deposition and can regulate cellular crosstalk. Kay EJ, et.al., 2022 [Ref 8], Schwörer S,et.al.,2021 [Ref 9], Pucino V, et.al., 2023 [Ref 10]

**Major point:**

 The proposed model at the end of the article is not very clear and can be improved.

Is the review written in accessible language?

Yes

Are all factual statements correct and adequately supported by citations?

Yes

Are the conclusions drawn appropriate in the context of the current research literature?

Partly

Is the topic of the review discussed comprehensively in the context of the current literature?

Partly

Reviewer Expertise:

Immunology, Immunometabolism, fibroblast biology, T cell biology, autoimmunity.

I confirm that I have read this submission and believe that I have an appropriate level of expertise to confirm that it is of an acceptable scientific standard, however I have significant reservations, as outlined above.

## References

[1] : Fibroblast heterogeneity: Keystone of tissue homeostasis and pathology in inflammation and ageing. *Front Immunol* .2023;14: 10.3389/fimmu.2023.1137659 1137659 10.3389/fimmu.2023.1137659 36926329 PMC10011104

[2] : Cross-tissue, single-cell stromal atlas identifies shared pathological fibroblast phenotypes in four chronic inflammatory diseases. *Med* .2022;3(7) : 10.1016/j.medj.2022.05.002 481-518.e14 10.1016/j.medj.2022.05.002 35649411 PMC9271637

[3] : Publisher Correction: Single-cell analysis uncovers fibroblast heterogeneity and criteria for fibroblast and mural cell identification and discrimination. *Nat Commun* .2020;11(1) : 10.1038/s41467-020-18511-8 4493 10.1038/s41467-020-18511-8 32883975 PMC7471259

[4] : Lymph node homeostasis and adaptation to immune challenge resolved by fibroblast network mechanics. *Nat Immunol* .2022;23(8) : 10.1038/s41590-022-01272-5 1169-1182 10.1038/s41590-022-01272-5 35882934 PMC9355877

[5] : A single cell atlas of frozen shoulder capsule identifies features associated with inflammatory fibrosis resolution. *Nat Commun* .2024;15(1) : 10.1038/s41467-024-45341-9 1394 10.1038/s41467-024-45341-9 38374174 PMC10876649

[6] : Microenvironmental sensing by fibroblasts controls macrophage population size. *Proc Natl Acad Sci U S A* .2022;119(32) : 10.1073/pnas.2205360119 e2205360119 10.1073/pnas.2205360119 35930670 PMC9371703

[7] : Mechanosensing regulates tissue repair program in macrophages. *Sci Adv* .2024;10(11) : 10.1126/sciadv.adk6906 eadk6906 10.1126/sciadv.adk6906 38478620 PMC10936955

[8] : Cancer-associated fibroblasts require proline synthesis by PYCR1 for the deposition of pro-tumorigenic extracellular matrix. *Nat Metab* .2022;4(6) : 10.1038/s42255-022-00582-0 693-710 10.1038/s42255-022-00582-0 35760868 PMC9236907

[9] : Fibroblast pyruvate carboxylase is required for collagen production in the tumour microenvironment. *Nat Metab* .2021;3(11) : 10.1038/s42255-021-00480-x 1484-1499 10.1038/s42255-021-00480-x 34764457 PMC8606002

[10] : Differential effect of lactate on synovial fibroblast and macrophage effector functions. *Front Immunol* .2023;14: 10.3389/fimmu.2023.1183825 1183825 10.3389/fimmu.2023.1183825 37304267 PMC10251493

# Reviewer report: Reviewer response for version 1

**DOI:** 10.5256/f1000research.157178.r262986

**Published:** 2024-05-07

**Authors:** Mateusz S. Wietecha

**Affiliations:** 1 University of Illinois Chicago, Chicago, Illinois, USA

In this review article, the authors describe the functions of fibroblasts in the context of ECM remodeling and their interaction with macrophages, and make the claim that CTHRC1 and TGFBI are important modulators of this axis. Overall, the manuscript is well-written. However, the manuscript is uneven in how it presents information. As I outline below, some sections of the manuscript are overly superficial in their discussion of important concepts and rely on citing review articles rather than primary research papers. Meanwhile, some of the other sections of the manuscript simply list findings from primary research studies without providing a proper context for the reader to understand the findings. The two main themes of fibroblast-macrophage interactions and CTHRC1/TGFBI functions read like two separate narratives, and the connection between the two needs to made much more clear by the authors.

 The following are a few suggestions to improve the manuscript:

 1. The title should be changed to reflect the rather narrow focus on either fibroblast-macrophage interactions and/or the roles of CTHRC1 and TGFBI.

 2. In general, the manuscript cites too many other reviews, especially when referring to specific facts/findings that should really be citing the original research papers that originated these findings.

 3. Intro, paragraphs 2-4, and section on "fibroblast heterogeneity dictates ECM deposition": the discussion about fibroblasts' heterogeneity and their myriad functions during wound repair and fibrosis is overly simplistic, as is the depiction in Figure 2. In addition to the discussed ref. 34, there are several other recent primary research papers that should be cited and discussed here that directly address fibroblast heterogeneity and its effects on ECM composition during wound repair; see for example references Guerrero-Juarez CF et.al., 2019 (Ref 1), Gay Det.al., 2020 (Ref 2), Foster DSet.al.,2021 (Ref 3), Wietecha MS et.al., 2023 (Ref 4), Phan QM et.al., 2021 (Ref 5), Hu KH et.al., 2023 (Ref 6) below. Many of these papers also mention fibroblast-macrophage interactions and list CTHRC1 and TGFBI as genes of interest, but this requires a deeper dive that may better set the stage for the more focused discussion about TGF-beta signaling, CTHRC1 and BIGH3.

 4. The sections on CTHRC1 and BIGH3 are a list of facts about their function in fibroblasts in various model systems and have very little to do with macrophages or fibroblast-macrophage interactions dictating ECM composition in disease states. Besides a couple sentences stating that both CTHRC1 and BIGH3 may also be produced by macrophages, how does this fit within the larger narrative the authors have established thus far? Are macrophages even the primary producers of these cofactors in disease states, or are fibroblasts? Indeed, the "proposed model for cofactor-mediated ECM deposition in fibroblasts" (Fig 4) does not show macrophages as producers of CTHRC1/BIGH3.

 5. The statement "This gene is also a paralog of periostin (POSTN)" lacks context and explanation.

Is the review written in accessible language?

Yes

Are all factual statements correct and adequately supported by citations?

Partly

Are the conclusions drawn appropriate in the context of the current research literature?

Partly

Is the topic of the review discussed comprehensively in the context of the current literature?

Partly

Reviewer Expertise:

wound healing, fibroblasts, bioinformatics

I confirm that I have read this submission and believe that I have an appropriate level of expertise to confirm that it is of an acceptable scientific standard, however I have significant reservations, as outlined above.

## References

[1] : Single-cell analysis reveals fibroblast heterogeneity and myeloid-derived adipocyte progenitors in murine skin wounds. *Nat Commun* .2019;10(1) : 10.1038/s41467-018-08247-x 650 10.1038/s41467-018-08247-x 30737373 PMC6368572

[2] : Phagocytosis of Wnt inhibitor SFRP4 by late wound macrophages drives chronic Wnt activity for fibrotic skin healing. *Sci Adv* .2020;6(12) : 10.1126/sciadv.aay3704 eaay3704 10.1126/sciadv.aay3704 32219160 PMC7083618

[3] : Integrated spatial multiomics reveals fibroblast fate during tissue repair. *Proc Natl Acad Sci U S A* .2021;118(41) : 10.1073/pnas.2110025118 10.1073/pnas.2110025118 34620713 PMC8521719

[4] : Phase-specific signatures of wound fibroblasts and matrix patterns define cancer-associated fibroblast subtypes. *Matrix Biol* .2023;119: 10.1016/j.matbio.2023.03.003 19-56 10.1016/j.matbio.2023.03.003 36914141

[5] : Single-cell transcriptomic analysis of small and large wounds reveals the distinct spatial organization of regenerative fibroblasts. *Exp Dermatol* .2021;30(1) : 10.1111/exd.14244 92-101 10.1111/exd.14244 33237598 PMC7839523

[6] : Transcriptional space-time mapping identifies concerted immune and stromal cell patterns and gene programs in wound healing and cancer. *Cell Stem Cell* .2023;30(6) : 10.1016/j.stem.2023.05.001 885-903.e10 10.1016/j.stem.2023.05.001 37267918 PMC10843988
